# Deep Brain Photoreceptor (*val-opsin*) Gene Knockout Using CRISPR/Cas Affects Chorion Formation and Embryonic Hatching in the Zebrafish

**DOI:** 10.1371/journal.pone.0165535

**Published:** 2016-10-28

**Authors:** Chong Yee Hang, Shogo Moriya, Satoshi Ogawa, Ishwar S. Parhar

**Affiliations:** Brain Research Institute, Jeffrey Cheah School of Medicine and Health Sciences, Monash University Malaysia, Bandar Sunway, Selangor, Malaysia; University of Hyderabad, INDIA

## Abstract

Non-rod non-cone photopigments in the eyes and the brain can directly mediate non-visual functions of light in non-mammals. This was supported by our recent findings on vertebrate ancient *long* (VAL)-opsin photopigments encoded by the *val-opsinA* (*valopa*) and *val-opsinB* (*valopb*) genes in zebrafish. However, the physiological functions of *valop* isoforms remain unknown. Here, we generated *valop*-mutant zebrafish using CRISPR/Cas genome editing, and examined the phenotypes of loss-of-function mutants. F0 mosaic mutations and germline transmission were confirmed via targeted insertions and/or deletions in the *valopa* or *valopb* gene in F1 mutants. Based on *in silico* analysis, frameshift mutations converted VAL-opsin proteins to non-functional truncated forms with pre-mature stop codons. Most F1 eggs or embryos from F0 female *valopa/b* mutants showed either no or only partial chorion elevation, and the eggs or embryos died within 26 hour-post-fertilization. However, most F1 embryos from F0 male *valopa* mutant developed but hatched late compared to wild-type embryos, which hatched at 4 day-post-fertilization. Late-hatched F1 offspring included wild-type and mutants, indicating the parental effects of *valop* knockout. This study shows *valop* gene knockout affects chorion formation and embryonic hatching in the zebrafish.

## Introduction

Extra-visual biological impacts of light (i.e. non-visual functions of light) are mediated by non-visual light detection systems. Our understanding of the complex mechanisms underlying mammalian non-visual photoreception could benefit from insights gained through studies of simplified non-mammalian model systems. In non-mammals, photoreception often involves extra-retinal photopigments that comprise light-absorbing chromophores bound to distinct G-protein coupled receptor opsins, such as those in the deep brain [1]. Recent studies have associated direct photoreception via deep brain photopigments with the control of seasonal reproduction in birds [2, 3] and the photic control of motor responses in zebrafish larvae [4–6].

Vertebrate ancient *long* (VAL)-opsin is a member of the deep brain opsin family and has been detected in the eyes and the brain of birds, reptiles, and fishes [7–14]. Two isoforms of zebrafish VAL-opsin, encoded by the *val-opsinA* (*valopa*) and *val-opsinB* (*valopb*) genes [11], exist and might have separate functions. Indeed, our recent studies concluded that VAL-opsin isoforms are involved in different neurochemical systems in multiple brain regions and mediate the control of time- and light-dependent physiology and behaviours in adult zebrafish [15, 16]. However, no reverse genetic studies have been conducted to study the functions of deep brain opsins including VAL-opsin in non-mammalian models. Although a knockdown study demonstrated the possible involvement of VAL-opsinA in the photic control of motor response in zebrafish embryos [4], the physiological functions of each VAL-opsin isoform in the adult zebrafish remain unknown.

Recently, clustered regularly interspaced short palindromic repeats/CRISPR-associated systems (CRISPR/Cas) emerged as a new tool for reverse genetic studies, particularly in non-model organisms. CRISPR/Cas can induce site-specific mutations with high efficiency and low off-target effects in vertebrates without the use of embryonic stem cells [17–19]. The CRISPR/Cas system was originally identified as a bacterial defence system against viral infections. This system inactivates viruses by inducing double-stranded DNA breaks in their genomic DNA using RNA-guided nucleases [20–22]. Adoption of the CRISPR/Cas system to generate mutant zebrafish [23–25] promises a rapid and effective platform to create a library of zebrafish genetic models for biomedical research. Moreover, the high efficiency of mutant generation indicates the potential for loss-of-function mutant analysis in the F0 generation [26, 27].

In the current study, mutant zebrafish harboring a modified *valop* gene were created using the CRISPR/Cas system, and phenotypes in F0 and F1 *valop* mutants were examined to propose physiological roles for VAL-opsin in zebrafish. This is the first knockout (KO) model to allow the functional analysis of a deep brain opsin in a non-mammalian vertebrate.

## Materials and Methods

### Animals

Adult wild-type zebrafish *Danio rerio* on the RIKEN Wako (rw) background were used in this study. RIKEN Wako (RW) wild-type strain was obtained from the Zebrafish National BioResource Center of Japan (http://www.shigen.nig.ac.jp/zebra/). Fish bred in our laboratory were maintained in freshwater aquaria at room temperature (27 ± 0.5°C) under a controlled lighting regime (14-h light/10-h dark). The fish were fed with adult zebrafish diet (Zeigler Bros., PA, USA) thrice daily. Larval fish were initially fed *Paramecium sp*. and were later fed live brine shrimp (*Artemia sp*.; the cysts from Zebrafish Management, UK) supplemented with ZM-100 Fry Food (Zebrafish Management, UK).

### Ethics statement

This study was carried out in strict accordance with the recommendations in the Guidelines to promote the wellbeing of animals used for scientific purposes: The assessment and alleviation of pain and distress in research animals (2008) by the National Health and Medical Research Council of Australia (https://www.nhmrc.gov.au/guidelines-publications/ea18). All experimental protocols were approved by the Animal Ethics Committee of Monash University (MARP-2012-146).

### Designs of guide RNA oligos

A published CRISPR/Cas system set-up protocol was followed, with slight modification [24]. The online software package ZIFIT Targeter version 4.2 (http://zifit.partners.org/ZiFiT/) was used to identify guide RNA (gRNA) target sites requiring the structure GG-(N)_18_-NGG. These gRNA sequences (Table 1) were selected for target exons of zebrafish *valopa* (GenBank accession number, NM_131586) and *valopb* (GenBank accession number, AY_996588). We selected gRNA sequences with no more than five predicted off-target sites and three mismatches on the zebrafish genome DNA database. The gRNA#1 and #2 sites were chosen for each *valop* isoform as a dual-CRISPR/Cas excision strategy to delete large (approximately 10 kb) DNA fragments spanning an intron. To contruct gRNA expression vectors, gRNAs, sense and anti-sense oligomers of the gRNAs were synthesized. A 5’-overhang TA- or AAAC- was included in each gRNA oligomer were to allow directional cloning into an expression vector.

**Table 1:** Target sites and sequences of guide RNAs.

Gene (Chr.)	gRNA target# (position)	Exon	Strand	Oligo sequence (5’–3’)
	5’–GG-(18 bp Target)-NGG–3’			
*valopa* (Chr.13)	gRNA#1 (+680)	4	–	TAGGCGTTACCCAGCCTGCCGT
	GGCGTTACCCAGCCTGCCGT**GGG**			AAACACGGCAGGCTGGGTAACG
	gRNA#2 (+1082)	5	–	TAGGATGTGGCTGAAAGTGCTG
	GGATGTGGCTGAAAGTGCTG**CGG**			AAACCAGCACTTTCAGCCACAT
*valopb* (Chr.12)	gRNA#1 (+631)	3	+	TAGGTGTGATCATTATCTCCTA
	GGTGTGATCATTATCTCCTA**TGG**			AAACTAGGAGATAATGATCACA
	gRNA#2 (+965)	5	–	TAGGTTCAGATTGGTGGATTCC
	GGTTCAGATTGGTGGATTCC**AGG**			AAACGGAATCCACCAATCTGAA

Positions of the *valopa/b* guide RNA (gRNA)#1 and #2 sites in different chromosomes (chr.) are relative to the ATG start codon. NGG (bold) represents the proto-spacer adjacent motif sequence. A 5’-overhang TA- or AAAC- was added to each gRNA oligomer.

### Production of guide RNAs and Cas9 mRNA

To construct the gRNA expression vector, pDR274 (plasmid # 42250; Addgene, Cambridge, MA, USA) was digested with the *BsaI* restriction enzyme. Next, a pair of gRNA oligonucleotides was annealed and cloned into the *BsaI*-digested pDR274 vector. Next, gRNAs were transcribed from *DraI*-digested gRNA expression vectors using the MEGAscript T7 Transcription Kit (Ambion, Austin, TX, USA), purified via ethanol precipitation, and re-dissolved in RNase-free water.

For Cas9 nuclease, the pT3TS-nCas9n expression vector used by Jao *et al*. (2013) was obtained from Addgene (plasmid # 46757). The Cas9 expression vector was linearized using the *XbaI* restriction enzyme. Using the *XbaI*-digested Cas9 plasmid as a template, the Cas9 mRNA was transcribed using mMESSAGE mMACHINE T3 ULTRA Transcription Kit (Ambion, Austin, TX, USA), a poly(A)-tailed with the poly(A)-Tailing kit (Ambion), treated with DNaseI, and purified using an RNeasy Micro Kit (QIAGEN, Hilden, Germany).

### Microinjection of embryos

A group comprising one male and two female zebrafish was placed in a tank in the evening. The next morning (30 min after lights on), their embryos were collected for microinjection. The embryos were lined along trenches (0.95 mm wide × 0.80 mm deep) made in a 1% agarose gel in a 100-mm diameter culture dish. Microinjection was performed using an Eppendorf Femtojet Microinjector (Eppendorf, Hauppauge, NY, USA), which was controlled using an InjectMan N2 micromanipulator (Eppendorf) under a stereoscopic microscope (SMZ-1000; Nikon, Tokyo, Japan). The gRNAs and Cas9 mRNA were co-injected into the yolks of one-cell stage embryos. Each embryo was injected with approximately 2 nl of solution containing 100 ng/μl of each gRNA and 150 ng/μl of Cas9 mRNA. The doses of gRNA and Cas9 mRNA were modified in accordance to the doses recommended in a protocol obtained from Wente and Chen group (http://www.addgene.org/crispr/Chen/). As each batch of embryos underwent injection, the remaining embryos were kept in a water-containing dish and placed on ice to delay cell division. After the injection, the F0 embryos were raised in clean water-containing petri dishes at 25 ± 1°C.

### Genotyping

Fish were anesthetized by immersion in a 0.01% solution of benzocaine (Sigma, St. Louis, MO, USA) prior to fin clipping. For genotype, a small part (~1 mm^2^) of the caudal fin was clipped and placed directly in a 25-μl PCR reaction containing a 1x concentration of TERRA PCR Direct Red Dye Premix (Clontech Laboratories, Mountain View, CA, USA) and 15 pmol of each PCR primer to amplify the *valopa/b* gRNA#1 and #2 sites. The PCR primer sets are listed in Table 2. The reaction program comprised the following steps: 98°C for 2 min and 40 cycles of 98°C for 10 s, 60°C for 15 s, and 68°C for 30 s, followed by 68°C for 7 min. The PCR products were purified using a DNA Clean and Concentrator-5 Kit (Zymo Research, Irvine, CA, USA) and subjected to direct sequencing using the BigDye Terminator v3.1 Cycle Sequencing Kit (Applied Biosystems, Woburn, MA, USA). Upon identification of mutant zebrafish, the ZFIN nomenclature guidelines (http://zfin.org/) were referenced for mutant line designation.

**Table 2:** PCR primers used for genotyping.

Gene	Nucleotide sequence (5’–3’)		Target site	Product size
*valopa*	Forward	TGACCCAGAGCATTTACAACACA	*valopa* gRNA#1	399 bp
	Reverse	ACGTTCCCACTGCACCCA		
	Forward	AGTAAAGTGACTGTGGTGGAAGGAA	*valopa* gRNA#2	184 bp
	Reverse	GGGACACACTTTGTTCTCAGGTATG		
*valopb*	Forward	TCTTTGACCCTCCTACCAATACCA	*valopb* gRNA#1	398 bp
	Reverse	ACGCAACTATCATTACAACGACCA		
	Forward	CTCTGATCCTTATTTACTTTCAATCTAACACTC	*valopb* gRNA#2	416 bp
	Reverse	CAATCCAATAGAGGTAACAACTGCTG		

The forward primer for *valopa/b* guide RNA (gRNA)#1 and the reverse primer for *valopa/b* gRNA#2 were paired to detect large DNA fragment deletions.

To estimate mutation frequency of each F0 fish, DNA amplicons containing the *valopa* or *valopb* gRNA#1 and gRNA#2 sites were cloned into a pGEM-T Easy vector (Promega, Madison, WI, USA). The plasmids containing a *valopa/b* DNA fragment from randomly selected E-coli colonies (N = 30) were purified using Wizard™ Plus SV Minipreps DNA Purification System (Promega); and were subjected to direct sequencing (provided by First BASE Laboratories Sdn. Bhd. Malaysia). Mutation frequencies (i.e. number of mutant clones/number of examined clones) were calculated for individual F0 fish.

### Determination of F1 mutant genotypes

Each of the F0 mutant fish was outcrossed with a wild-type (WT) partner. The F1 embryos were raised to adulthood and selected for genotyping via fin clipping, as described above. Actual insertion and/or deletion (in-del) mutations at the *valopa/b* gRNA#1 and #2 sites were confirmed through sequencing analysis. Mutation rates (i.e. number of F1 mutants at the gRNA#1 site/ number of selected F1 fish) were initially calculated. The F1 fish selected for genotyping were male dominant. The F1 fish including those found to harbor mutations at the *valopa/b* gRNA#1 site were further checked for mutations at the *valopa/b* gRNA#2 site (mutation rates, i.e. number of F1 fish mutants at the gRNA#2 site/number of initially screened F1 fish). The ratios of frameshift mutation and in-frame mutation at the *valopa/b* gRNA#1 and #2 sites were also determined.

### *In silico* analysis of mutant proteins

Based on the confirmed genotypes, amino acid sequences of mutant VAL-opsinA and VAL-opsinB were deduced using software GENETYX version 8.0 (GENETYX Co., Tokyo, Japan). Next, the number of transmembrane domains was predicted using the online program TMHMM Server version 2.0 (http://www.cbs.dtu.dk/services/TMHMM-2.0/). The bovine rhodopsin model [28] was referenced in the analysis of conserved functional motifs in opsins.

### Examination of mutant phenotypes

Sexually mature F0 mutant fish (approximately 3 months old) were examined regarding morphology, swimming behaviour, and breeding capability. The fish were monitored daily. We noticed an unexpected phenotype in the eggs and embryos obtained from F0 *valop* mutant breeding. Accordingly, follow-up experiments were conducted using available F0 adult fish of each gender and with mutations in each *valop* isoform.

To examine F0 female fish, a breeding tank for the mating of a female mutant or a WT sibling with a male WT partner was set up in the evening. The following morning, F1 eggs were collected in a petri dish containing clean water. For the comparison purposes, breeding groups of two F0 female mutants and a WT sibling were set up at the same time. The numbers of dead eggs or embryos (opaque appearance) were recorded at 2 or 3 hour-post-fertilization (hpf) and 26 hpf to calculate their mortality rates (number of dead eggs/total number of spawned eggs). Images of the eggs or embryos were captured at 4 hpf under a stereoscopic microscope (SMZ-1500, Nikon) equipped with a digital camera (D5-2Wv, Nikon). Embryonic hatching rates were also recorded, as described below.

To examine F0 male fish, the same breeding method was used to mate a male mutant or a WT sibling with a female WT partner. Breeding of a F0 male mutant and a WT sibling was set up simultaneously. Embryonic mortality rates were recorded as described above. The numbers of embryos hatched at 3 day-post-fertilization (dpf) and 4 dpf were recorded (3 hours after lights on) to calculate hatching rates (numbers of hatched embryos/total number of survived embryos at 3 dpf). Images of the embryos were captured at 0 dpf, 4 dpf, and 6 dpf as described above.

### Statistical Analysis

Endpoint data (number of dead/not or number of hatched/not) observed in F1 offspring (eggs/embryos) from F0 mutant and wild-type sibling parents was compared using a contingency table. To evaluate whether our hypothesis is likely to be true that the targeted mutations in F0 parents is associated with the number of dead eggs/embryos and hatched embryos, a Chi-Square test was performed in Microsoft Excel 2013 with level of statistical significance set at p < 0.01. To further determine that the *valop* mutation in F0 male and female parents (mutant/not) is a dependent factor for their offspring phenotype (dead/not and hatch/not), a Logistic Regression test was performed using online program (http://statpages.info/logistic.html).

## Results

### Generation of *valop*-mutant zebrafish

**Fig 1:**
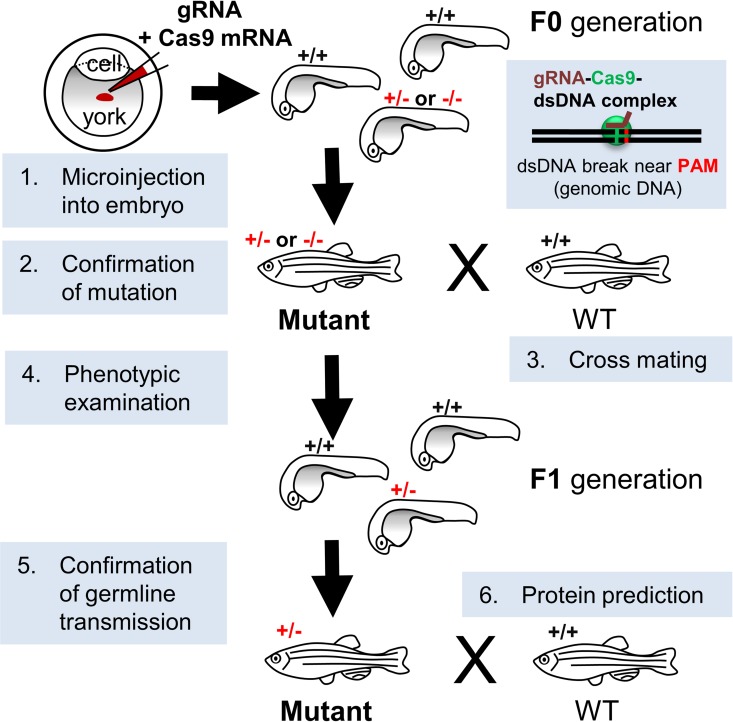
A flowchart summarizing the generation of mutant zebrafish using the CRISPR/Cas system. (1) microinjection of gRNAs and the Cas9 mRNA into embryos, (2) confirmation of mutation by DNA sequencing, (3) mating of an identified mutant (+/- or -/-, red) with a wild-type (+/+) partner, (4) phenotypic examination, (5) confirmation of germline transmission in the F1 generation, and (6) *in silico* protein prediction. According to the CRISPR/Cas concept, gRNA (brown line) and Cas9 nuclease (green circle) complex with genomic DNA (black double lines) at the gRNA target site near PAM (red dots on the double lines) to cleave dsDNA, leading to in-del mutations as a result of non-homologous end joining DNA repair. Abbreviation: CRISPR/Cas, clustered regularly interspaced short palindromic repeats/CRISPR-associated systems; dsDNA, double-stranded DNA; gRNA, guide RNA; PAM, proto-spacer adjacent motif.

The procedure used to generate *valop* mutant zebrafish is shown in Fig 1. The *valop* mutants were created by the injection of Cas9 mRNA and gRNA customized to target the *valop* isoform genes. During the screening of the injected F0 embryos, no large DNA fragment deletions of the *valop* genes spanning both the gRNA#1 and #2 sites in the *valop* genes were detected in our attempts. Therefore, we proceeded to check individual *valopa/b* gRNA target sites to detect in-del mutations.

**Fig 2:**
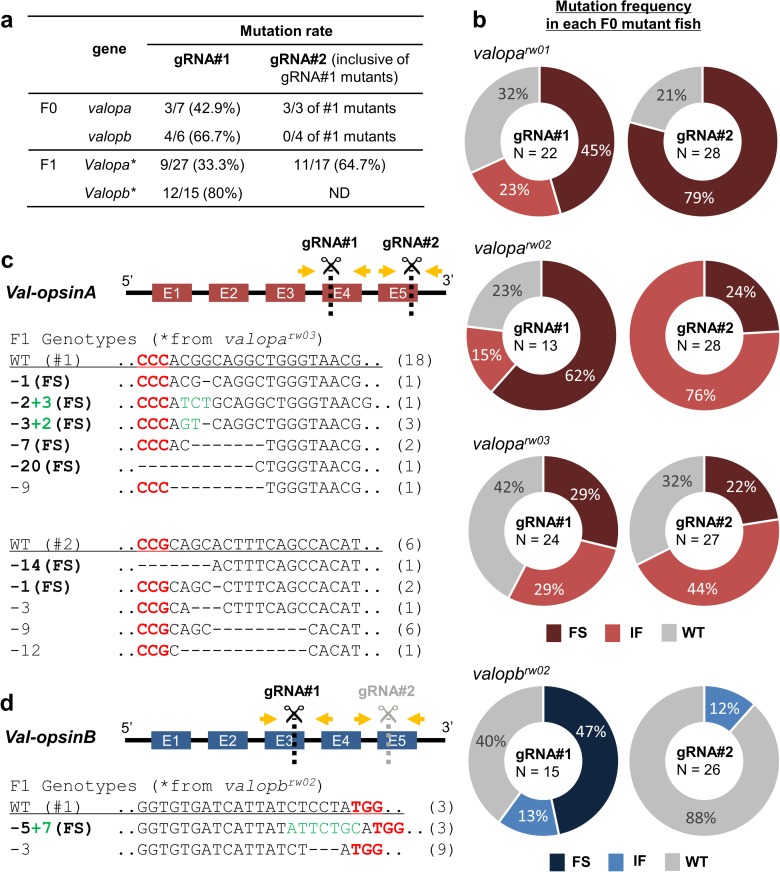
Heritable mutations of the *val-opsin* isoform genes. (**a**) Table for mutation rates at the *valopa/b* gRNA#1 and #2 sites in the F0 generations and the F1 offspring (*) derived from representative F0 *valopa*^*rw03*^ and *valopb*^*rw02*^ mutants. (**b**) Pie charts for mutation frequencies of individual F0 fish, i.e. *valopa*^*rw01*^, *valopa*^*rw02*^, *valopa*^*rw03*^, and *valopb*^*rw02*^ mutants, indicate different ratio of cloned DNAs with a wild type (WT), in-frame (IF), or frameshift (FS) genotype. Schematic drawings of the exons of the *val-opsinA* (**c**) and *val-opsinB* exons (**d**) indicate the DNA cleavage positions (dotted lines) with the guide RNA (gRNA)#1 and #2; and the PCR primer positions (yellow arrows) for genotyping. Details of the F1 offspring genotypes are provided below. The F1 genotypes contain deletions (dashes) and insertions (green letters) at the *valopa/b* gRNA#1 and #2 sites (black letters) near proto-spacer adjacent motif sequences (red letters). The numbers of deleted or inserted base pairs and resultant frameshift (FS) mutations are indicated. The numbers of mutants obtained with each genotype are shown in parentheses. Note that a F0 mutant with no detectable mutations at the *valopb* gRNA#2 site was used to produce F1 offspring (ND, not determined). N represents number of bacterial clones containing a target DNA.

In the *valopa* gene, three out of seven F0 fish had mutations at the gRNA#1 site, and all of the three also had mutations at the gRNA#2 site (Fig 2A). In the *valopb* gene, four out of six F0 fish had mutations at the gRNA#1 site; however, none of the four identified mutants had detectable mutations at the gRNA#2 site (Fig 2A). A clear mixture of DNA sequencing chromatograms starting at the *valopa/b* gRNA target sites was observed, indicating mosaic mutations in the genome of the *valop* mutant genome. The first six F0 mosaic *valop* mutants were named *valopa*^*rw01*^, *valopa*^*rw02*^, *valopa*^*rw03*^, *valopb*^*rw01*^, *valopb*^*rw02*^, and *valopb*^*rw03*^.

F0 fish with no detectable mutations at the *valopa/b* gRNA#1 site were considered as WT siblings in this study. However, mutations at the *valopa/b* gRNA#2 site in these WT siblings remained undetermined, because this factor might not be critical (normal phenotype in embryos from the WT siblings was observed).

Analysis of bacterial clones containing a target DNA from F0 mutant fish showed each of which had different levels of genetic mosaicism, i.e. a mixture of wild-type, in-frame- and frameshift-mutated genotypes. Most of the clones from F0 fish, i.e. *valopa*^*rw01*^, *valopa*^*rw02*^, and *valopa*^*rw03*^ mutants, had mutations at the gRNA#1 site (57–77%; N = 13–24) and gRNA#2 site (79–100%; N = 27–28) (Fig 2B). Most of the clones from F0 *valopb*^*rw02*^ mutant, had mutations at the gRNA#1 site (60%; N = 15) (Fig 2B); but the *valopb* gRNA#2 site had a low mutation frequency (12%; N = 26), which was not detectable with clipped fin tissue.

After the F1 offspring from representative F0 *valopa*^*rw03*^ and *valopb*^*rw02*^ mutants reached adulthood, inheritable mutations and exact genotypes were confirmed. In the F1 offspring from *valopa*^*rw03*^ mutant, 17 fish underwent genotyping of both the *valopa* gRNA#1 and gRNA#2 sites. Four fish had no detectable mutations, four fish had mutations at both the *valopa* gRNA#1 and #2 sites, and nine fish had single mutations: two at the *valopa* gRNA#1 site, and seven at the gRNA#2 site (Fig 2A).

In the F1 offspring from *valopb*^*rw02*^ mutant, 12 out of 15 fish had mutations at the *valopb* gRNA#1 site (Fig 2A). We did not examine mutations at the *valopb* gRNA#2 site in the F1 offspring, because the *valopb*^*rw02*^ mutant had no detectable mutations at the *valopb* gRNA#2 site.

Among the F1 offspring from a single F0 mutant, a variety of in-del mutations were detected at the *valop* gRNA#1 and #2 sites (Fig 2C and 2D). The ratios of frameshift to in-frame mutations were eight to one at the *valopa* gRNA#1 site, and three to eight at the *valopa* gRNA#2 site (Fig 2C); and a three to nine at the *valopb* gRNA#1 site (Fig 2D).

### Modifications in VAL-opsin proteins

Some *valopa* and *valopb* mutants possess frameshift mutated VAL-opsin proteins, which lacked critical functional motifs for opsin function according to a study of bovine rhodopsin [28], as the result of the identified in-del mutations at the *valopa/b* gRNA#1 site.

**Fig 3:**
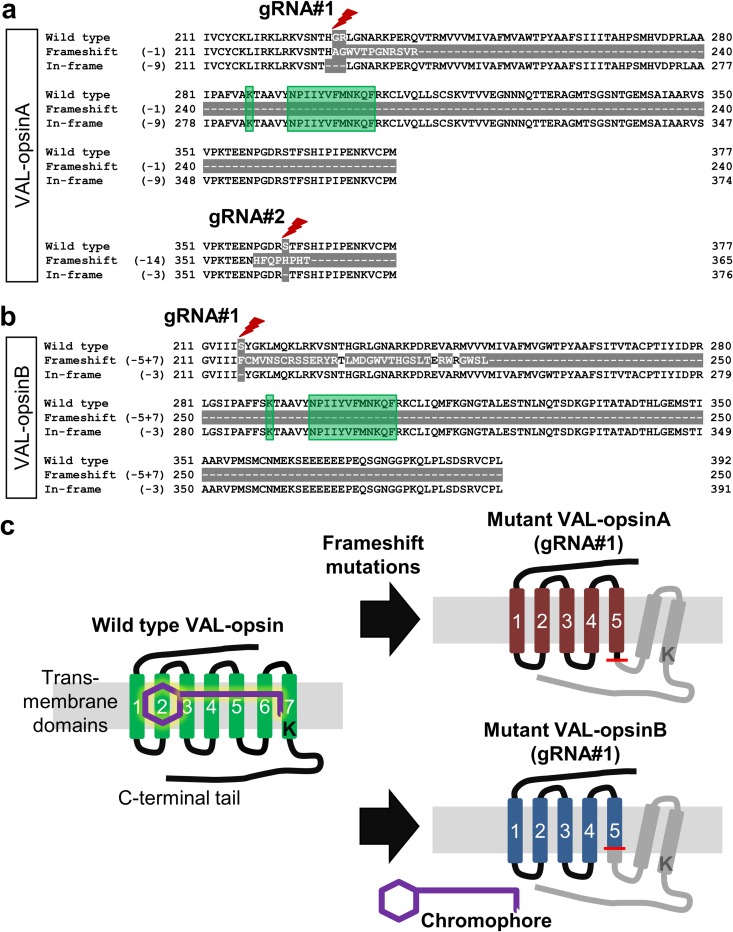
Deduced amino acid sequences of the VAL-opsin proteins from representative mutants. Alignments of partial peptide sequences indicating truncated peptide parts (grey highlights), targeted mutation sites (red bolts), and opsin-specific functional motifs such as the lysine K residue in the seventh transmembrane domain and the NPxxY(x)5,6F motif (green highlights) of VAL-opsinA (**a**) and VAL-opsinB (**b**). (**c)**, Schematic drawing of the structures of wild-type VAL-opsin (*left*; transmembrane domains in green), and the truncated mutant VAL-opsins (*right*; transmembrane domains in red or blue) resulting from frameshift mutations (red lines) with premature stop codons. Accordingly, the VAL-opsin proteins resulting from frameshift mutations at the gRNA#1 site lack the K residue that binds to the light-absorbing chromophores (purple); and are rendered non-functional.

Representative frameshift and in-frame mutations at the *valopa* gRNA#1 site (-1 bp and -9 bp) and the *valopb* gRNA#1 site (–5+7 bp and –3 bp) and their predicted amino acid coding sequences are shown in Fig 3. Frameshift mutations at the gRNA#1 site in *valopa* and those in *valopb* introduced premature stop codons into the intracellular loop between the transmembrane domain (TM) 5 and TM6, and in the TM5, respectively, which led to truncated five-transmembrane VAL-opsinA and VAL-opsinB (Fig 3A and 3B). Other frameshift mutants also exhibited disrupted amino acid sequences after the TM5. The disrupted region contains functional VAL-opsin motifs, such as the lysine residue at position 287 or 291 in the TM7 and the NPxxY(x)5,6F domain in the carboxyl (C)-terminal tail. As a result, the VAL-opsin proteins resulting from frameshift mutation at the gRNA#1 site lacked these functional motifs, and thus were virtually unable to form functional photopigments (Fig 3C).

In contrast, the in-frame mutations at the gRNA#1 site introduced deletions of one and three amino acids into VAL-opsinA and VAL-opsinB, respectively (Fig 3A and 3B). Similar amino acids deletions in the VAL-opsin proteins occurred in other in-frame mutants. These in-frame gRNA#1 site mutations maintained VAL-opsins with the seven transmembrane domains and the functional motifs.

Representative frameshift and in-frame mutations (-14 bp and -3 bp) among the obtained mutations at the *valopa* gRNA#2 site are shown in Fig 3A. Frameshift mutation led to a mutated VAL-opsinA with a slightly shorter C-terminal tail and altered amino acid sequence (Fig 3A). In-frame mutation led to VAL-opsinA that lacked a single amino acid in the C-terminal tail (Fig 3A). Similar modifications in the VAL-opsinA amino acid sequence were detected in other frameshift or in-frame mutants. These gRNA#2 site-mutant VAL-opsinA proteins maintained the seven transmembrane domains and the functional motifs.

### Effects of *valop* KO in zebrafish

We next examined the KO phenotypes of the F0 *valop* mutants. The F0 mutant zebrafish maintained morphological features and breeding capabilities and were indistinguishable from WT siblings (gross observation). Note that the WT siblings were injected with gRNAs and Cas mRNA but failed to effectively incur mutations at the *valopa/b* gRNA#1 site, similar to embryos injected with a non-targeting gRNA (S1 File). Normally upon spawning, the chorion envelope in the egg is elevated, and the blastodisc in the fertilized egg expands and develops into embryonic cells in the subchorionic space. Most of the embryos hatch at 4 day-post-fertilization (dpf). However, we observed significant abnormalities in the eggs/embryos from F0 female mutants and the resultant embryos from F0 male mutants.

**Fig 4:**
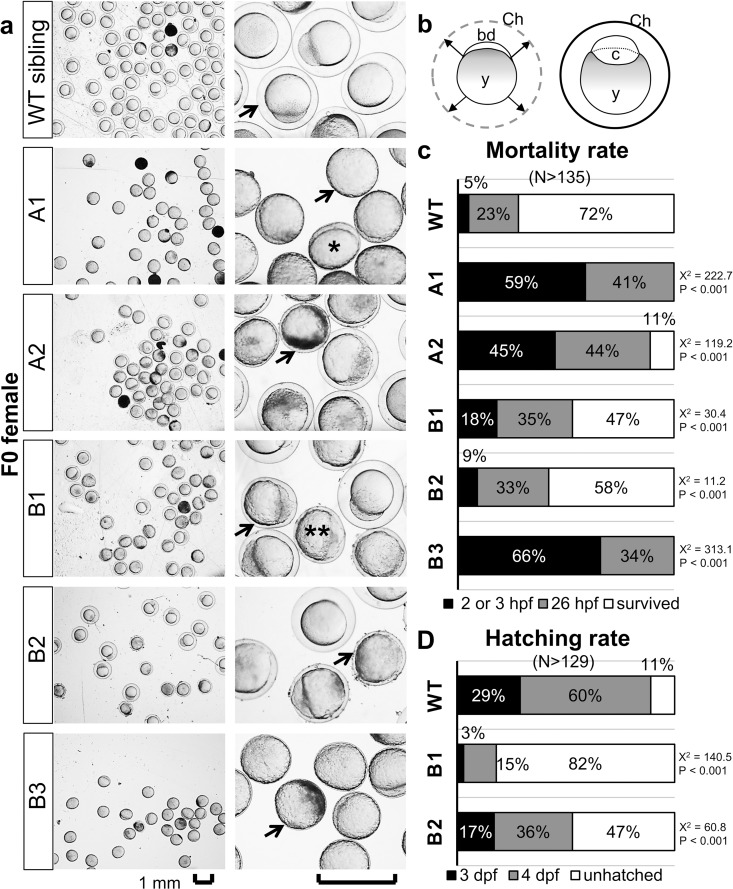
Abnormal chorion elevation in the eggs or embryos obtained from F0 female *valopa/b* mutants. (**a)** Images of zebrafish eggs or embryos obtained at 4 hour-post-fertilization (hpf) from a female WT sibling and female mutants: *valopa*^*rw01*^ (represented by A1), *valopa*^*rw02*^ (A2), *valopb*^*rw01*^ (B1), *valopb*^*rw02*^ (B2), *valopb*^*rw03*^ (B3). Upon spawning, chorion is elevated from the surface of the egg, and the blastodisc develops into embryonic cells in the created space (**b**). Arrows indicate the chorion surrounding the egg. Note that the eggs or embryos obtained from the *valopa/b* mutants exhibited no (single asterisk) or only partial (double asterisks) chorion elevation. Eggs with partial chorion elevation exhibited blastodisc expansion; although some develop into embryos, all eventually died (opaque appearance). (**c** and **d**) The mortality rates of the eggs or embryos and the hatching rates of embryos, respectively. Embryos from B1 and B2 mutants survived but mostly failed to hatch at 4 day-post-fertilization (dpf), in contrast to most embryos from the WT sibling hatched. Note that the association between the *valop* mutations in individual F0 parents and the increased number of dead eggs/embryos at 26 hpf and the decreased number of hatched embryos at 4 dpf was statistically significant (determined by Chi-Square X^2^ values and p-values). Logistic Regression analysis showed that the observed phenotype is likely dependent on the targeted mutation (data in S1 File). N represents the number of eggs or embryos; and percentages shown in a single bar add up to a total of 100%. Abbreviation: bd, blastodisc; c, embryonic cells; Ch, chorion; y, yolk. Scale bar, 1 mm.

Mating of female *valopa*^*rw01*^, *valopa*^*rw02*^, *valopb*^*rw01*^, *valopb*^*rw02*^, and *valopb*^*rw03*^ mutants with a random male WT partner produced eggs with either no or only partial chorion elevation (Fig 4). In eggs with no chorion elevation, blastodisc retained as a small size and crescent shape. In contrast, eggs with partial chorion elevation exhibited blastodisc expansion, however, their embryonic development was disturbed by the limited subchorionic space (Fig 4A and 4B). The abnormal eggs or embryos eventually died (indicated by opaque appearance).

In the current study, eggs or embryos obtained from *valopa*^*rw01*^ and *valopa*^*rw02*^ mutants exhibited mortality rates of 59% (number of eggs, N = 204) and 45% (N = 135) at 2 hpf, respectively; at 26 hpf, the mortality rates increased to 100% and 89%, respectively (Fig 4C). In contrast, eggs or embryos from *valopb*^*rw01*^ and *valopb*^*rw02*^ mutants had mortality rates of 18% (N = 337) and 9% (N = 433) at 3 hpf, respectively, and 53% and 42% at 26 hpf, respectively (Fig 4C). Similar to the *valopa*^*rw01*^ or *valopa*^*rw02*^ mutants, eggs or embryos from the *valopb*^*rw03*^ mutant had mortality rates of 66% (N = 324) at 3 hpf, and 100% at 26 hpf (Fig 4C). In contrast, the embryos from the WT sibling had low mortality rates of 5% (N = 199) at 2 hpf and 28% at 26 hpf (Fig 4C).

Embryos from *valopb*^*rw01*^ and *valopb*^*rw02*^ mutants exhibited somewhat normal chorion elevation, with survival rates of 47% and 58%, respectively. However, surviving embryos from the *valopb*^*rw01*^ and *valopb*^*rw02*^ mutants had low hatching rates of 18% (N = 127) and 53% (N = 236) at 4 dpf, respectively (Fig 4D). However, 74% (N = 125) and 97% (N = 234) of the embryos from the respective mutants had hatched at 5 dpf. In contrast, 89% (N = 122) of embryos from the WT sibling hatched at 4 dpf. Genotyping revealed that most surviving embryos from the *valopb*^*rw02*^ mutant were also mutants (80%; Fig 2A).

**Fig 5:**
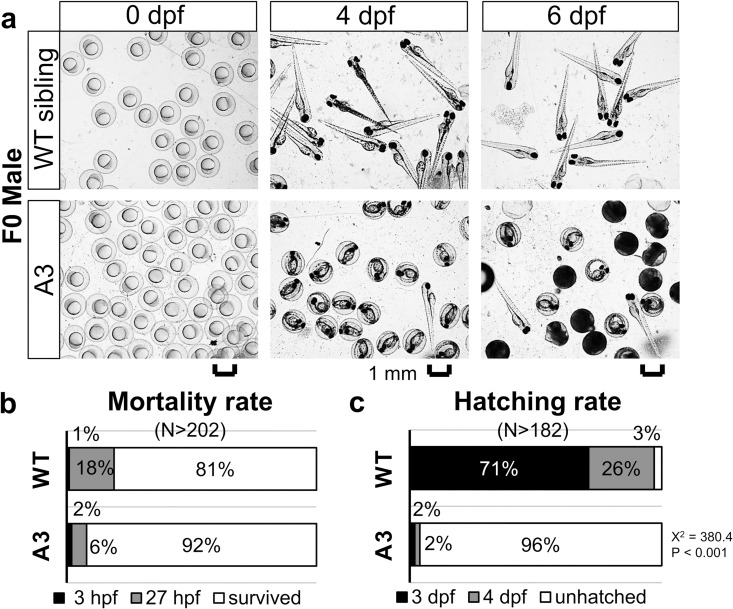
Late-hatching embryos obtained from a F0 male *valopa* mutant. (**a)** Images of the zebrafish embryos obtained from a male WT sibling and a male mutant *valopa*^*rw03*^ (represented by A3) at 0 day-post-fertilization (dpf), 4 dpf, and 6 dpf. (**b** and **c)** the mortality rates of the eggs or embryos and hatching rates of the embryos, respectively. Most embryos from the mutant had not hatched at 4 dpf, in contrast to most embryos from the WT sibling hatched. At 6 dpf, most of the unhatched embryos had died in the chorion. Genotyping of F1 adult offspring identified a mixture of WT and mutants. Note that the association between the *valop* mutations in individual F0 parents and the decreased number of hatched embryos at 4 dpf was statistically significant (determined by Chi-Square X^2^ values and p-values). Logistic Regression analysis showed that the observed phenotype is likely dependent on the targeted mutation (data in S1 File). N represents the number of eggs or embryos; and percentages shown in a single bar add up to a total of 100%. Scale bar, 1 mm.

Mating of a male *valopa*^*rw03*^ mutant with a random female WT partner resulted in late-hatching embryos (Fig 5). These eggs exhibited normal chorion elevation and normal embryos development (Fig 5A). By 27 hpf, embryos from the mutant showed a low mortality rate (8%, N = 202), in comparison with embryos from the WT sibling (19%, N = 316) (Fig 5B). However, only 4% (N = 182) of embryos from the *valopa*^*rw03*^ mutant hatched on the normal hatching day (4 dpf), in contrast to 97% (N = 255) of embryos from the WT sibling (Fig 5B). Most embryonic offspring from the male mutant (83%, N = 202) eventually died by 6 dpf without hatching. However, 17% of embryos from this mutant hatched at later time points. Genotyping confirmed the presence of some mutants (33.3%) among F1 adult offspring of the *valopa*^*rw03*^ mutant (Fig 2A). Note that F0 male fish harboring *valopb* mutations was not available.

The association between the *valop* mutations in individual F0 parents and the increased number of dead eggs/embryos at 26 hpf (from female *valopa*^*rw01*^, *valopa*^*rw02*^, *valopb*^*rw01*^, *valopb*^*rw02*^, and *valopb*^*rw03*^ mutants) was statistically significant (large Chi-Square values ranged from 11.2 to 313.1 with one degree of freedom, and p < 0.001). Similarly, the effect on the decreased number of hatched embryos at 4 dpf (from male *valopa*^*rw03*^, and female *valopb*^*rw01*^ and *valopb*^*rw02*^ mutants) was statistically significant (large Chi-Square values ranged from 60.8 to 380.4 with one degree of freedom, and p < 0.001). According to Logistic Regression analysis, the odds of eggs/embryos obtained from F0 female *valopa* mutants and *valopb* mutants die at 26 hpf were high (Odds Ratio values 51. 550 and 4.078, respectively, meaning 51 times and 4 times greater chance; p < 0.001; data in S1 File). Moreover, the odds of embryos resulted from F0 male *valopa* mutant and female *valopb* mutants hatch at 4 dpf were low (Odds Ratio values 0.001 and 0.043, respectively, meaning only 0.1% and 4.3% chance; p < 0.001; data in S1 File). Therefore, our hypothesis is most likely to be true that the *valop* mutations in F0 parents affect the number of dead eggs/embryos and the number of hatched embryos.

## Discussion

To our knowledge, this is the first reverse genetics study of a deep brain opsin based on genetic KO in a non-mammalian model. Using the CRISPR/Cas system, targeted in-dels were introduced into the *valopa* or *valopb* genes in zebrafish, resulting in F0 mosaic mutants. DNA sequencing of the F1 generation confirmed mutations resulting in non-functional VAL-opsin proteins. The F0 fish harboring mosaic mutations of the *valop* isoforms produced eggs or embryos with developmental abnormalities. These abnormalities were observed in most F1 embryos, regardless of mutation, suggesting the effects of *valop* KO in the F0 generation.

### Loss-of-function mutations

In the current study, mutations at either the *valopa or valopb* gRNA#1 site (but not both isoforms) were detected in nearly half of the injected fish in the F0 generation. Furthermore, the F0 *valopa* mutants also harbored mutations at the *valopa* gRNA#2 site. These results confirmed the high efficiency of the CRISPR/Cas-mediated mutagenesis in the zebrafish. Because large DNA fragment deletions were not detected in *valop* genes in the present study, further optimizations is likely needed to achieve a high rate of large DNA fragment deletion. As we will discuss later, the modifications at the *valopa/b* gRNA#1 site were sufficient to generate a loss of VAL-opsin function in mosaic mutants.

In the current study, we observed phenotypic changes in the F0 generation. CRISPR/Cas has been used to generate the sufficient appearance of KO phenotypes in the F0 generation via targeted mutation in vertebrates, e.g. zebrafish [26], Atlantic salmon [29], tilapia [30], and mice [31, 32]. In the present study, DNA amplicons containing the *valopa* or *valopb* gRNA#1 site from individual F0 fish, i.e. *valopa*^*rw01*^, *valopa*^*rw02*^, *valopa*^*rw03*^, and *valopb*^*rw02*^ mutants, showed high mutation frequencies (58–77%). Consistent with this, additional results from a single injected embryo also showed high mutation frequencies at the *valopa/b* gRNA#1 site (4 out of 5 clones). The high efficiency of mutagenesis estimated for individual F0 fish and the phenotypic changes in the F0 generation suggest (bi-allelic) mutation, particularly at the *valop* gRNA#1 site, in a substantial number of the cells.

The CRISPR/Cas genome editing generally yields low off-target mutation rates [33–36]. Although we cannot neglect the possibility of off-target mutation, the consistent phenotypic alteration in several independent *valopa/b* mutants indicates the effect of the targeted KO, rather than off-target mutations.

CRISPR/Cas-targeted modifications of the *valop* genes were germline transmitted, resulting in F1 heterozygous mutants. According to the genotyping of the F1 generation, the *valopa*^*rw03*^ germlines had a mutation rate of 77% at either the *valopa* gRNA#1 site or the gRNA#2 site; and those of *valopb*^*rw02*^ mutant had a mutation rate of 80% at the *valopb* gRNA#1 site. The identification of more than two genotypes in the F1 generation from a single F0 mutant indicates germline mosaicism in the F0 mutants. Germline mosaicism is the result of the DNA cleavage and repair at multicellular stages, because 32-cell-stage embryos (1.5 hpf) already contain four primordial germ cells [37, 38]. Separate CRISPR/Cas activity might occur in these germ cells, as the CRISPR/Cas components were injected into the yolk. This issue could be overcome via injection into the one-cell-stage embryonic cell.

Frameshift mutations at the *valopa/b* gRNA#1 site disrupted the amino acid sequence beyond the TM5, resulting in truncated VAL-opsins that lacked crucial functional motifs. However, neither in-frame mutations at the gRNA#1 sites nor mutations at the gRNA#2 sites critically altered the VAL-opsin motifs. Based on *in silico* analyses, frameshift-induced truncated VAL-opsins lacked a conserved lysine residue in the TM7 domain, which binds the chromophores [28]; a conserved NPxxY(x)5,6F motif for receptor structural dynamics [39, 40]; and the TM6 and the carboxyl-terminal cytoplasmic tail, which transmit input light to induce biochemical signalling [41, 42]. In a cell culture model of a blind cavefish, the transfection with the zebrafish genes encoding melanopsin or teleost-multiple-tissue opsin, but not with the genes encoding opsins truncated at the TM5 or TM6, conferred light sensitivity upon otherwise light-insensitive cells [43]. Notably, zebrafish VA-opsin, a shorter intrinsic genetic variant of VAL-opsinA that lacked most of the carboxyl-terminal tail, did not exhibit spectral absorbance *in vitro* [10]. These findings indicate the functional significance of the region disrupted in the current study. In the present study, at least 89% of the F1 mutants from *valopa*^*rw03*^ and 25% of the F1 mutants from *valopb*^*rw02*^ carried one frameshift-mutated *valop* gene allele encoding for a non-functional protein.

### Parental-effect KO phenotypes

Six F0 *valopa/b* mutants, most of which expressed non-functional VAL-opsin, appeared morphologically normal and were capable of breeding. No apparent abnormal behaviours were observed. Therefore, *valop* gene mutations in zebrafish might currently have little or no effects on the body development, sexual maturation and behaviour.

The most apparent phenotype observed in eggs from F0 female *valopa/b* mutants was the lack of or partial chorion elevation. Most of these abnormal eggs failed to develop into embryos. Eggs that exhibited development at certain stages eventually died, likely due to the limited subchorionic space for development. Moreover, approximately half the eggs from *valopa*^*rw01*^, *valopa* ^*rw02*^, and *valopb*^*rw03*^ mutants had died by 2 or 3 hpf, suggesting a major failure of oocyte maturation in the ovary. Failure to ovulate has been observed in female zebrafish exposed to diethylstilbestrol (a non-steroid estrogen) or testosterone-containing water, and eggs artificially collected from these fish do not exhibit chorion elevation [44], a hallmark of egg activation. Similar abnormal chorion elevation was observed in the eggs from female zebrafish harboring mutations in maternal genes: including two undefined genes [45, 46] and one gene required for a calcium-dependent egg activation signal [47]. Indeed, genotyping of surviving embryos from *valopb*^*rw01*^ and *valopb*^*rw02*^ mutants revealed a mixture of wild-type and mutant offspring. Therefore, the KO of the *valop* genes is proposed to cause maternal effects on the oocytes of female zebrafish, resulting in a failure of oocyte maturation or egg activation.

Embryos from F0 male *valopa*^*rw03*^ mutant developed normally, but hatched at a later time point, compared with offspring from a male WT sibling. Although only one male *valopa* mutant was available for examination, late hatching was also observed in the surviving embryos from F0 female *valopb*^*rw01*^ and *valopb*^*rw02*^ mutants. Interestingly, recent studies showed that ambient light conditions affect zebrafish hatching; specifically, light deprivation and the exposure to red light delay hatching of WT embryos [48, 49]. Therefore, we expected that non-functional VAL-opsin was the cause of delayed hatching of the F1 embryos. However, subsequent genotyping of hatched embryos from the *valopa*^*rw03*^ mutant revealed that the late-hatching embryos are indeed a mixture of wild-type and mutant offspring. This finding indicates that the late embryonic hatching was due to a parental effect. Interestingly, late-hatching embryos can be obtained from zebrafish and medaka breeders exposed to endocrine disrupting drugs such as ibuprofen [50, 51] and diclofenac [52]. Although both males and females were exposed to the drugs in those studies, the results suggest that embryonic hatching is affected by the parental conditions. In fact, the gene expression of several reproductive hormones such as gonadotropin-releasing hormone, follicle-stimulating hormone, and luteinizing hormone was disrupted in male and female zebrafish exposed to ibuprofen [51]. Taken together, the KO of the *valop* genes in zebrafish is proposed to delay embryonic hatching due to a mutation-related parental hormonal control on the embryo.

Parental effects resulting from *valopa/b* KO are also supported by a comparison between the number of eggs or embryos exhibiting the abnormalities and the mutation rate in the F1 generation. Nearly all F1 eggs or embryos from three of the female *valopa/b* mutants exhibited abnormal chorion elevation and later died. In contrast, less than half of the eggs or embryos from female *valopb*^*rw02*^ mutant died, as a result of abnormal chorion elevation. Most F1 adult offspring from this mutant were found to harbor mutations at the gRNA#1 site. In addition, almost all F1 embryos from the male *valopa*^*rw03*^ mutant hatched late, a third was found to harbor mutations at the gRNA#1 site. Accordingly, the number of F1 eggs or embryos with the abnormalities did not correlate with the mutation rate in the F1 generation. F0 WT siblings with no detectable mutations at the *valop* gRNA#1 site had a normal phenotype, even though some might have harbored mutations at the *valopa* gRNA#2 site. Therefore, we concluded that the F0 *valop*-KO zebrafish exhibited a parental-effect phenotype.

In the present study, *valop* gene mutation was predicted to functionally affect VAL-opsin proteins throughout the body. In the zebrafish, the VAL-opsin isoforms are expressed in both the eyes and the brain [10, 11, 15], and the VAL-opsin photoreceptors in the eyes and the brain are thought to play distinct roles in zebrafish physiology [16]. Furthermore, VAL-opsinA and VAL-opsinB photoreceptors are expected to have distinct functions in the eyes [11]. In the current study, F0 *valopa* and *valopb* mutants exhibited a similar parental-effect phenotype in their eggs and resultant embryos. Our previous work determined the co-expression of *valopa* and *valopb* in the thalamic *valop* cell group within the brain, which may play a GABA-dependent modulatory role in light-dependent physiology [15, 16]. There is evidence of seasonal variation in the levels of GABA-synthesizing enzymes in the brain and GABA control on pituitary gonadotropin release in fish species [53–57]. Taken together, the lack of functional VAL-opsin in the brain, particularly in the thalamus, is likely to account for the parental-effect phenotype observed in the current study.

The current *valop*-KO zebrafish model revealed promising parental effects in the F0 generation, although limitations were apparent. First, we were unable to examine the exact genotype resulting in the KO phenotype in mosaic *valop* mutants. Second, the small number of available F0 *valop* mutants limited the number of biological replicates for detailed phenotypic analysis. Nonetheless, the observed different degrees of KO phenotype are likely due to the levels of mosaic KO in individual F0 *valop* mutants. Future studies will examine the genotype-phenotype link and underlying mechanisms in homozygous *valop* mutants.

**Fig 6:**
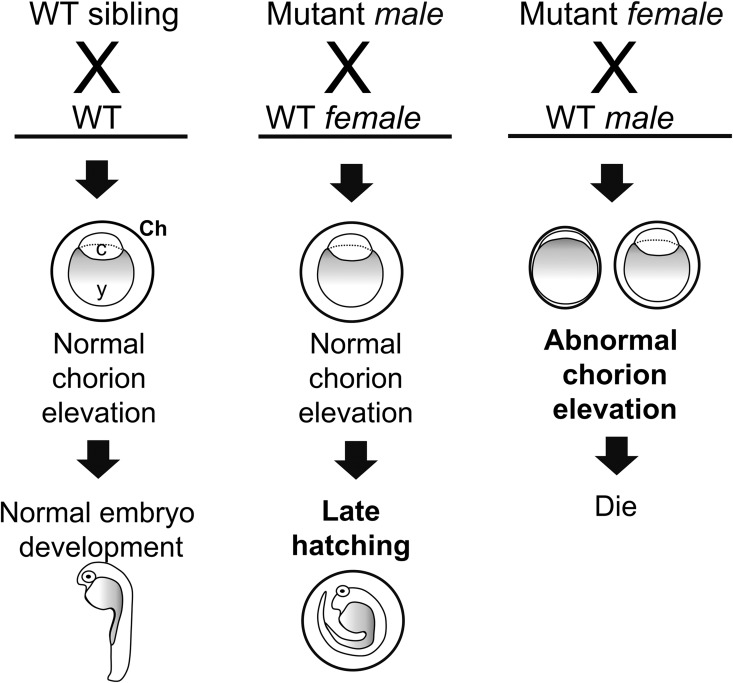
An overview of the parental effects of *valop* KO in the zebrafish. Mating of F0 *valopa/b* mutants (male or female) with a WT partner resulted in F1 eggs or embryos exhibiting abnormalities (bold letters). Genotyping of the F1 offspring from *valopa*^*rw03*^ and *valopb*^*rw02*^ mutants indicate a mixture of WT and mutants and suggest the parental effects of *valop* KO. The parental-effect phenotypes observed in the current study is shared by the *valopa* and *valopb* mutants. This suggests that *valopa* and *valopb* co-expressing cells in the brain might be involved in the control of zebrafish reproduction. Abbreviation: c, embryonic cells; Ch, chorion; KO, knockout; y, yolk.

In summary, this is the first genetic analysis of a deep brain opsin in a non-mammalian model, as a basis for further functional studies of VAL-opsins in the zebrafish. As illustrated in Fig 6, F0 female breeders expressing mosaic *valopa/b* KO produced eggs with abnormal chorion elevation and these eggs or resultant embryos eventually died. Moreover, mating of a F0 male breeder harboring a mosaic *valopa* KO with a WT female resulted in late hatching embryos. These abnormalities were also observed in WT embryos from the F0 mutants, suggesting the parental effects of *valop* KO. Therefore, the VAL-opsin photoreceptors might play a reproductive role in zebrafish. The complicated photic regulation of fish reproduction has been previously documented [58–60]; and the current findings indicate new research directions for further studies.

## Supporting Information

S1 File Effect of co-injection of Tbait gRNA and Cas9 mRNA on *valopa* and *valopb* gene target sequences and Logistic Regression analysis of the *valopa/b* mutations in F0 parent as a dependent factor for mortality of eggs/embryos (female) or embryonic hatching (male) (DOCX)
