# Supplementary material for: Deep Brain Photoreceptor (val-opsin) Gene Knockout Using CRISPR/Cas Affects Chorion Formation and Embryonic Hatching in the Zebrafish
Source: PLoS One. 2016 Oct 28;11(10):e0165535. doi: 10.1371/journal.pone.0165535 (PMC5085036; doi:10.1371/journal.pone.0165535)
Supplement: S1 File — (DOCX) [file pone.0165535.s001.docx]

S1 File

**Deep brain photoreceptor (*val-opsin*) gene knockout using CRISPR/Cas affects chorion formation and embryonic hatching in the zebrafish**

Chong Yee Hang, Shogo Moriya, Satoshi Ogawa, and Ishwar S. Parhar*

*** Corresponding author:**

Email: [ishwar@monash.edu](mailto:ishwar@monash.edu) (ISP)

S1 Text

As a negative control, we used the gRNA that complements to a 23 bp sequence derived from the mouse Tet1 gene, Tbait [reported in Wang et al. (2013)].

In the current study, Tbait gRNA and Cas9 mRNA was injected into one-cell stage embryos. At 5 day-post-fertilization, 5 eggs were randomly selected and their genomic DNAs were isolated and the sequences of target regions in *valopa* and *valopb* genes were examined by direct sequencing. The methodology was the same as described in main text. Our result (S1 Sequence Data) showed that the *valop* gRNA regions were not affected by Tbait gRNA injection.

**S1 Reference**

1. Wang, H, Yang H, Shivalila CS, Dawlaty MM, Cheng AW, Zhang F, Jaenisch R. One-step generation of mice carrying mutations in multiple genes by CRISPR/Cas-mediated genome engineering. Cell. 2013; 153, 910–918. PubMed PMID: 23643243.

S1 Sequence Data

*valopa* gRNA#1

1 ACCTGACGCTCCGGTTTCCTGGCGTTACCCAGCCTGCCGTGGGTGTTGGAGACCTAAACA

2 ACCTGACGCTCCGGTTTCCTGGCGTTACCCAGCCTGCCGTGGGTGTTGGAGACCTAAACA

3 ACCTGACGCTCCGGTTTCCTGGCGTTACCCAGCCTGCCGTGGGTGTTGGAGACCTAAACA

4 ACCTGACGCTCCGGTTTCCTGGCGTTACCCAGCCTGCCGTGGGTGTTGGAGACCTAAACA

5 ACCTGACGCTCCGGTTTCCTGGCGTTACCCAGCCTGCCGTGGGTGTTGGAGACCTAAACA

************************************************************

*valopa* gRNA#2

1 CACTTTGTTCTCAGGTATGGGGATGTGGCTGAAAGTGCTGCGGTCTCCAGGATTTTCCTC

2 CACTTTGTTCTCAGGTATGGGGATGTGGCTGAAAGTGCTGCGGTCTCCAGGATTTTCCTC

3 CACTTTGTTCTCAGGTATGGGGATGTGGCTGAAAGTGCTGCGGTCTCCAGGATTTTCCTC

4 CACTTTGTTCTCAGGTATGGGGATGTGGCTGAAAGTGCTGCGGTCTCCAGGATTTTCCTC

5 CACTTTGTTCTCAGGTATGGGGATGTGGCTGAAAGTGCTGCGGTCTCCAGGATTTTCCTC

************************************************************

*valopb* gRNA#1

1 CATGCTTTATCCTTCCTCTTGGTGTGATCATTATCTCCTATGGTAAACTCATGCAGAAGC

2 CATGCTTTATCCTTCCTCTTGGTGTGATCATTATCTCCTATGGTAAACTCATGCAGAAGC

3 CATGCTTTATCCTTCCTCTTGGTGTGATCATTATCTCCTATGGTAAACTCATGCAGAAGC

4 CATGCTTTATCCTTCCTCTTGGTGTGATCATTATCTCCTATGGTAAACTCATGCAGAAGC

5 CATGCTTTATCCTTCCTCTTGGTGTGATCATTATCTCCTATGGTAAACTCATGCAGAAGC

************************************************************

*valopb* gRNA#2

1 CGGCCCTTTGTCTGATGTCTGGTTCAAATTGGTGGATTCCAGGGCCGTGCCGTTTCCTTT

2 CGGCCCTTTGTCTGATGTCTGGTTCAAATTGGTGGATTCCAGGGCCGTGCCGTTTCCTTT

3 CGGCCCTTTGTCTGATGTCTGGTTCAAATTGGTGGATTCCAGGGCCGTGCCGTTTCCTTT

4 CGGCCCTTTGTCTGATGTCTGGTTCAAATTGGTGGATTCCAGGGCCGTGCCGTTTCCTTT

5 CGGCCCTTTGTCTGATGTCTGGTTCAAATTGGTGGATTCCAGGGCCGTGCCGTTTCCTTT

************************************************************

S1 Tables

Results were described in main text. Here, we showed data of the statistical analyses.

1. **Logistic Regression analysis of the *valopa* mutations in F0 female parent as a dependent factor for mortality of eggs/embryos**

| Variable | Coefficients | Standard Errors | p value | Odds Ratios | 95% Confidence Limits | |
| --- | --- | --- | --- | --- | --- | --- |
|  |  |  |  |  | Low | High |
| Mutant | 3.9426 | 0.3007 | 0.000 | 51.5502 | 28.5916 | 92.9444 |

Data categorized as mutant or not mutant and dead or not dead were analyzed.

1. **Logistic Regression analysis of the *valopb* mutations in F0 female parent as a dependent factor for mortality of eggs/embryos**

| Variable | Coefficients | Standard Errors | p value | Odds Ratios | 95% Confidence Limits | |
| --- | --- | --- | --- | --- | --- | --- |
|  |  |  |  |  | Low | High |
| Mutant | 1.4415 | 0.1576 | 0.000 | 4.227 | 3.032 | 5.8931 |

Data categorized as mutant or not mutant and dead or not dead were analyzed.

1. **Logistic Regression analysis of the *valopa* mutations in F0 male parent as a dependent factor for embryonic hatching**

| Variable | Coefficients | Standard Errors | p value | Odds Ratios | 95% Confidence Limits | |
| --- | --- | --- | --- | --- | --- | --- |
|  |  |  |  |  | Low | High |
| Mutant | -6.7864 | 0.5436 | 0.000 | 0.0011 | 0.0004 | 0.0033 |

Data categorized as mutant or not mutant and hatched or not hatched were analyzed.

1. **Logistic Regression analysis to predict the *valopb* mutations in F0 female parent as a dependent factor for embryonic hatching**

| Variable | Coefficients | Standard Errors | p value | Odds Ratios | 95% Confidence Limits | |
| --- | --- | --- | --- | --- | --- | --- |
|  |  |  |  |  | Low | High |
| Mutant | -3.1383 | 0.4036 | 0.000 | 0.0434 | 0.020 | 0.0956 |

Data categorized as mutant or not mutant and hatched or not hatched were analyzed.
